# Heat stress effects on offspring compound across parental care

**DOI:** 10.1098/rspb.2025.0026

**Published:** 2025-03-05

**Authors:** Tanzil Gaffar Malik, Mu-Tzu Tsai, Benjamin James Mervyn Jarrett, Syuan-Jyun Sun

**Affiliations:** ^1^ National Taiwan University, Taipei, Taiwan; ^2^ School of Natural Sciences, Bangor University, Bangor, UK

**Keywords:** climate change, plasticity, heat stress, reciprocal transplant, burying beetle, parental care

## Abstract

Heatwaves associated with climate change threaten biodiversity by disrupting behaviours like parental care. While parental care may buffer populations from adverse environments, studies show mixed results, possibly due to heat stress affecting different care components. We investigated how heat stress impacts parental care and offspring performance in the burying beetle *Nicrophorus nepalensis* under control (17.8°C) and heat stress (21.8°C) conditions. We focused on two critical periods: pre-hatching care (carcass preparation) and post-hatching care (offspring provisioning). To disentangle the vulnerability of these parental care components to heat stress, we reciprocally transferred carcasses prepared under control or heat stress to females breeding under both conditions. Heatwaves affecting only one care period did not alter reproduction, but when both pre- and post-hatching periods experienced heatwaves, reproductive success declined. Females exhibited higher energy expenditure during provisioning, evidenced by greater body mass loss. Notably, heat stress had long-lasting effects on offspring via carcass preparation, resulting in smaller adult size and higher mortality. These results highlight the complexity of environmental stressors on parental care, suggesting that different care components may respond differently to heat stress, and thus need to be examined separately to better understand how parental care responds to, and buffers against, temperature stress.

## 1. Introduction

Global climate change poses a significant threat to biodiversity, affecting ecosystems through rising temperatures and more frequent extreme weather events [1,2]. One of the most significant changes associated with climate change is the increased frequency and intensity of heatwaves [3]. Heatwaves are defined as periods of localized heat accumulation during consecutive days and nights of abnormally high temperatures [4]. These heatwaves can cause disruptions of life cycle and physiological stress, especially for ectotherms like insects, which are particularly vulnerable to fluctuations in ambient temperature, directly impacting their physiology and behaviour [5–7].

The timing of heatwaves can disproportionately impact species if it aligns with a particularly sensitive period [8], like that of providing parental care [9,10]. For instance, exposure to extreme temperatures during reproductive stages may impair key parental behaviours, leading to increased offspring mortality and reduced fitness [8,11]. Altricial offspring tend to be more vulnerable to environmental impacts, needing aid in thermoregulation, especially considering nests cannot be moved to cooler microhabitats as adults can [12]. Parents can help buffer their offspring from the negative effects of heat stress by plastically altering their suite of parental care behaviours. Nest-building birds can alter the structure and composition of the nests they build to provide a more resilient thermal environment in which their offspring develop [13–15]. After hatching, parents can also increase the number of provisioning visits to mitigate heat stress [16,17]. In many cases, however, heat stress negatively impacts parental care behaviours, at a severe cost to the parents and to their offspring; parents may reduce their levels of care to preserve and reallocate resources to their own survival [18], and increased provisioning may not even prevent population decline [18].

Parental care is composed of a suite of behaviours that may or may not respond to heat stress in the same manner, or equally buffer offspring from the negative effects of heat stress to the same degree [19,20]. For example, nesting birds build a nest before any chick hatches and can shape the nest according to local conditions, like temperature [21]. But for species that exhibit less plasticity in the shape and form of their nests, or use tree-holes [22], parents can only buffer offspring from temperature with their interactions after hatching, which may mean the impact of heat stress is greater in these species. Only by decomposing parental care into distinct components of care, can we obtain a greater mechanistic understanding of the impact of temperature on parental care behaviours, and be able to predict which behaviours are most beneficial for parental and offspring fitness and to identify which behaviours could evolve to facilitate persistence in a rapidly changing environment.

Here, we investigate the effects of elevated temperatures on two periods of parental care and subsequent offspring performance in *Nicrophorus nepalensis*. The burying beetles, *Nicrophorus* spp., exhibit elaborate parental care, including the preparation of carcasses as a food source for their offspring (pre-hatching care), as well as direct feeding behaviour provided to their young (post-hatching care [23,24]). These behaviours are crucial for the development and survival of the young [25] but are potentially vulnerable to thermal stress. As ectotherms, burying beetles expend energy at a rate determined by ambient temperature. Because parents and offspring feed from a shared carcass resource, thermal stress may amplify conflict over care by increasing not only the energetic demands [5,26] of both parents and offspring but also the rate at which energy is expended on behaviours such as carcass maintenance, provisioning and begging. Additionally, thermal stress may impair parental coordination or timing of parental care behaviours, further affecting offspring development and survival [25]. Previous studies have indicated that elevated temperatures can disrupt parental care behaviours in burying beetles, leading to suboptimal carcass preparation and reduced reproductive success [27]. However, Grew *et al*. [28] demonstrated that parental effects were beneficial but were temperature dependent. It was only under non-stressed conditions (15°C) that the presence of females enhanced larval survival, with no clear evidence of parental buffering at higher temperatures (20 and 25°C) [28]. Collectively, these findings highlight consistent negative impacts of heat stress on fitness, while also suggesting that the capacity of parents to mitigate these effects may vary depending on the severity of thermal stress.

**Figure 1.**
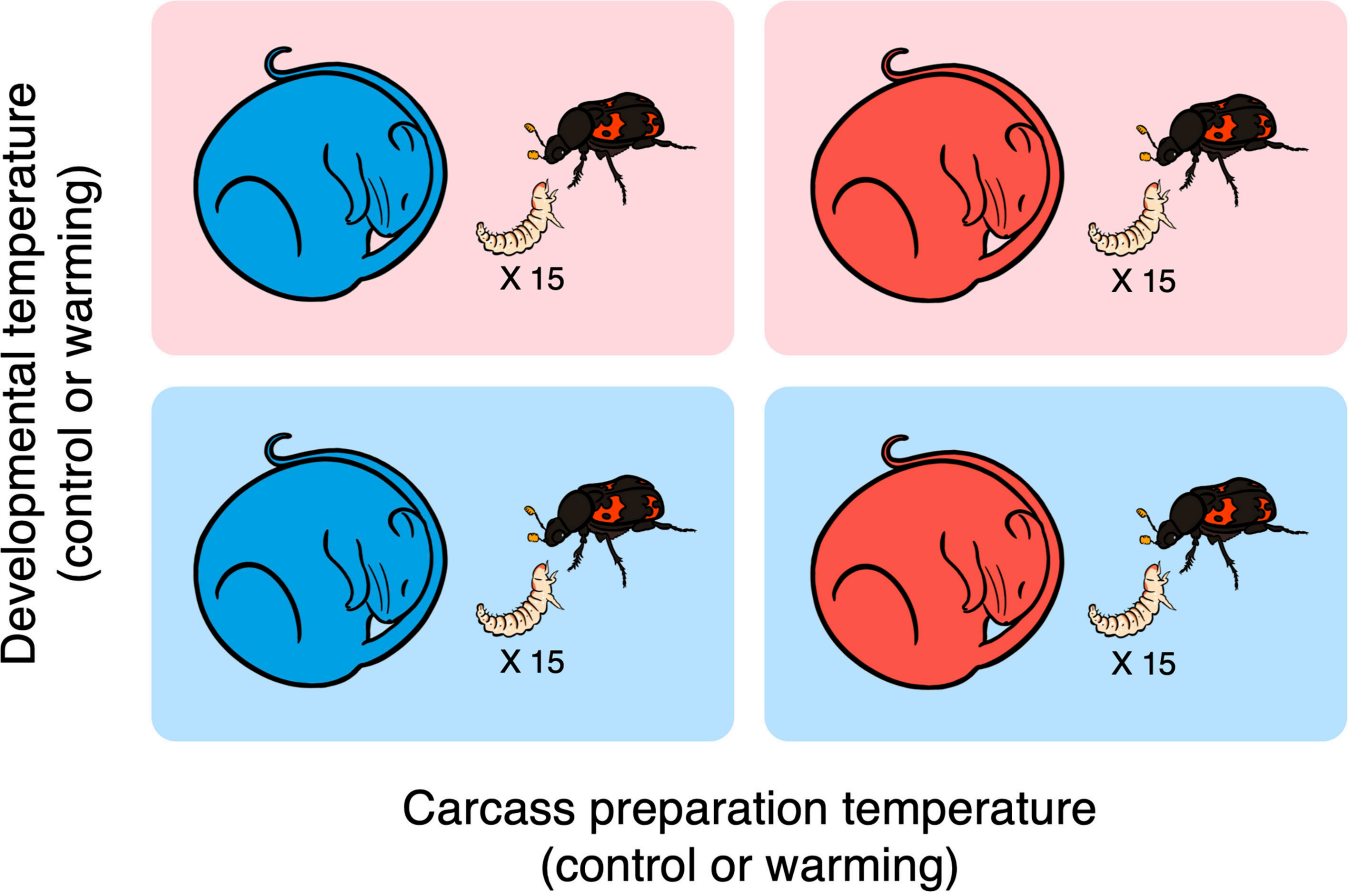
Experimental set-up of the reciprocal transplant experiment testing the joint and independent effects of temperatures (blue: control; red: warming) associated with carcass preparation and larval development. Females that had prepared carcasses at control temperatures were provided with either a carcass prepared by another beetle at control temperatures or warming temperatures. Fifteen newly hatched first larvae collected haphazardly from different families were introduced as a group of genetic mix.

These results highlight that there is an urgent need to investigate how exactly climate change influences parental care and thus provide insight into the potential mechanisms by which parents might buffer their offspring against rising and more extreme temperatures. We focus on how temperature-induced changes in carcass preparation by parents, along with direct temperature effects, affect the growth and survival of their larvae. In the first experiment, we evaluated the reproductive investment by exposing female beetles to control (17.8°C) and heat stress (21.8°C) conditions during pre-hatching care. We investigated (i) whether heat stress reduced the clutch size, (ii) whether heat stress makes carcass preparation more energetically costly, and (iii) whether heat stress disrupts the carcass preparation process. In the follow-up experiment, we used a reciprocal transplant experiment that transferred carcasses prepared under either control or heat stress conditions to control-reared beetles during breeding under both control and heat stress conditions (figure 1). This design allows us to investigate (i) the relative importance of carcass preparation and larval developmental temperature on reproductive success and (ii) whether parental care, partitioned as pre-hatching and/or post-hatching care, could mitigate the adverse impacts of heat stress. Our study seeks to provide insights into the potential adaptive responses of burying beetles to climate change and contribute to a broader understanding of how environmental stressors impact parental care.

## 2. Material and methods

### (a) Study system

*Nicrophorus nepalensis* is a subsocial beetle that exhibits biparental care, which is essential for the development and survival of offspring [29]. Parental care in this species consists of two primary stages: pre-hatching care and post-hatching care. During pre-hatching care, parents prepare a small vertebrate carcass as a resource for their offspring by removing fur or feathers and shaping the carcass into a compact ball. This process reduces decay and microbial activity prior to larval hatching [30]. In the post-hatching stage, parents actively feed and protect the larvae until they disperse [23,24]. Under natural temperature conditions during the beetle’s breeding season in northern Taiwan, carcass preparation typically lasts 3–4 days, while larval development lasts over 7–8 days [29]. The entire period of parental care thus lasts approximately 10–12 days under typical environmental conditions [29,31,32].

Heatwaves, characterized by short-term but significant increases in temperature, may selectively disrupt specific stages of parental care, such as carcass preparation and/or larval development. Field observations at our study sites revealed that the average duration of heat stress events was 4.21 days (median: 4 days), and in some cases, the heat stress persisted for 10–13 days (see electronic supplementary material, figure S1). These durations overlap with the timing of critical parental care activities. Thus, our experimental manipulation of heat stress in only one of the parental care stages (see below) aligns well with the field conditions typically experienced by beetles, where heat stress events are defined (see electronic supplementary material) as the days when the daily mean temperature exceeds 21.8°C.

### (b) Establishment of *Nicrophorus nepalensis* colony in the laboratory

*Nicrophorus nepalensis* beetles used in this experiment were descendants of a laboratory colony established in 2022. The source beetles were collected from six different locations in northern Taiwan (24.99° N, 121.62° E). Before breeding, all mites were removed from the beetles using fine tweezers. The beetles were housed individually in plastic individual boxes (10.8 × 7.5 × 2.1 cm) filled with moist soil and were fed minced pork (~1.5 g) twice a week. Two weeks after acclimation to laboratory conditions, we bred pairs of male and female *N. nepalensis* collected from different sites, provisioning them with 22–30 g of frozen mice (*n* = 60 pairs). We bred the beetles in cylindrical breeding boxes (14.2 × 6.3 cm) filled with 2 cm of moist soil. At larval dispersal, we determined brood size by counting all third-instar larvae, which were then transferred to eclosion boxes (2 × 2 × 2 cm) filled with moist soil and housed individually until reaching adulthood. The beetles were raised in controlled laboratory settings with a 10:14 light-to-dark cycle, 70% relative humidity and a daily temperature regime that mimicked the variations typical of their breeding season from November to April in northern Taiwan (mean: 17.8°C, with daily variations between 16°C and 20°C [33]). Each year, field beetles were collected and bred into the laboratory colony to maintain genetic diversity.

### (c) Effects of temperatures on pre-hatching care

The experiments were conducted in May–June 2024 using the laboratory-maintained beetle colonies. In *Nicrophorus* spp., both parents typically participate in the preparation of the carcass and care of the offspring, yet the males often prioritize seeking additional mating opportunities and so are likely to leave the females alone to take care of the broods [24]. We focused our experiments on females (because they can breed independently once mated) to avoid any confounding effects from male participation since males could influence the reproductive fitness by competing with females over carcass resources. To generate mated females, we paired up 118 males and females haphazardly. Each pair was placed in an individual box filled with moist soil and kept in an incubator set to 17.8°C as described above.

The next day, the males from each pair were removed, and only the mated females were transferred to breeding boxes filled with 2 cm of moist soil and provisioned with 23–29 g (25.82 ± 1.55 g; mean ± s.d.) of frozen mice (weighed to the nearest 0.01 g). Prior to introduction, we also weighed each female to the nearest 0.0001 g. These breeding set-ups were then incubated under two different temperature regimes: control and warming. The warming treatment temperature was elevated by 4°C (21.8°C) compared with the control (17.8°C), representing a projected extreme temperature increase by the end of this century. During breeding attempts, the boxes were kept in complete darkness to simulate underground conditions [34]. In total, there were 78 control and 40 warming treatment breeding attempts.

Ninety hours later, we determined clutch size and carcass preparation for each brood. At this stage, egg laying was largely completed, and the carcass was fully prepared (i.e. fur removal and rolling into a ball [29]). This timeline was chosen based on our prior observations, ensuring consistency in carcass preparation across different temperature treatments despite potential accelerated degradation or egg development at higher temperatures [29]. We counted the number of eggs that were visible at the bottom of the container [34,35]. This approach allows for an accurate proxy for exact total egg production in *N. nepalensis* [*36*]. The prepared carcass and the female were carefully removed and weighed again. The change in beetle and carcass mass allowed us to estimate the extent of carrion consumption during the carcass preparation stage. To determine the extent of carcass preparation, we took photos of each prepared carcass using two identical cameras (TG7-OM System). The carcasses were photographed against a white background from both the side and the top, with each camera placed approximately 30 cm away from the carcass. We determined the roundness of the carcass by taking the average values derived from the two images, using ImageJ (see [37,38] for details).

### (d) Experimental manipulation of carcass preparation and developmental temperatures

To investigate the temperature effects of carcass preparation and larval development, we used a reciprocal transplant experiment that transferred carcasses prepared at different temperatures (hereafter carcass preparation temperature) for control-reared beetles to breed upon at different temperatures (hereafter developmental temperature, i.e. the temperature experienced by larvae post-hatching). The carcasses, prepared at either control or warming temperatures from the previous experiment, were placed inside new breeding boxes filled with 2 cm of moist soil. These were set aside for later use in rearing experimental broods (see below). Since this study focuses on the effect of temperatures on carcass preparation and larval development, females that prepared carcasses at warming temperatures were not used to avoid the carry-over effect of warming. Thus, we initially allowed extra females to prepare carcasses in the control environment to ensure a sufficient number of females for the experiment. Each female was randomly assigned to one of the four experimental treatments. Importantly, females and carcasses from the same source were never assigned together. The treatments combined two factors: carcass preparation temperature (control or warming) and developmental temperature (control or warming). Hence, for example, carcasses prepared under warming conditions could only be assigned to treatments where developmental temperature was either control or warming. Introducing females that had prepared carcasses and laid eggs under control conditions also ensured that the beetles were of similar physiological conditions in the middle of reproduction prior to the transfer.

When the egg started hatching at 96 h, different broods of newly hatched larvae from carcasses prepared at control conditions were pooled in a Petri dish, 15 larvae were haphazardly selected and transferred to their designated experimental treatments using fine tweezers. We chose 15 individuals because this number falls within the brood range exhibited in our laboratory and field individuals when bred in the laboratory (ranging between 1 and 34, 14.7 ± 9.6, *n* = 60). This pool of unrelated larvae reduces potential confounding maternal effects such as sib-sib genetic influences and ongoing maternal care provided to the same genetic offspring, although it does not remove all maternal effects (e.g. maternal mRNAs that are potentially already present in eggs) [39]. The larvae were haphazardly assigned to one of the four experimental treatments, depending on the combination of carcass preparation temperature (C) and developmental temperature (D), with each treatment having control (control) and warming (warm) conditions: C_control_ – D_control_ (*n* = 21), C_warm_ – D_control_ (*n* = 17), C_control_ – D_warm_ (*n* = 20) and C_warm_ – D_warm_ (*n* = 20) (figure 1). There is no evidence that burying beetle parents can recognize kin, but rather, acceptance is contingent on larvae arriving within the expected time window from oviposition [31]. The larvae used in the experiment were time-matched with the expected hatch date of the focal female’s eggs to ensure acceptance by the females and to standardize developmental timing across all treatments. The larvae were placed gently on each carcass simultaneously. The boxes were then returned to their designated developmental temperatures. We checked the development of larvae daily from day 8 and recorded the day of larval dispersal into the nearby soil. At this stage, the entire brood was counted and weighed to the nearest 0.001 g using an analytical balance (Shimadzu, model ATX224R). Subsequently, all females were removed from the boxes, their body mass measured again and their size was estimated by measuring the pronotum width (to the nearest 0.01 mm) with a vernier calliper. The change in body mass has been used widely as an indicator of the cost associated with parental investment [40]. Following these measurements, the dispersing larvae were individually placed and incubated in eclosion boxes under control conditions. Upon eclosion, we determined if, and when, the offspring successfully eclosed as new adults, as evidenced by the complete transition of elytra colour from brown to black. Unsuccessful ones would die as larvae, pupae or even adults. For the successfully eclosed beetles, we determined their body size by measuring the pronotum width to the nearest 0.01 mm.

### (e) Statistical analysis

All statistical analyses were performed in R v. 4.1.2, with figures generated using the package ‘ggplot2’ [41]. Generalized linear mixed models (GLMMs) were performed with the *glmer* function in the ‘lme4’ package [42]. We checked for normal distribution of model residuals using the ‘DHARMa’ package [43]. This required us to use the negative binomial distribution for analysing clutch size and to log transform brood mass prior to analysis since our initial diagnostics indicated that the data were overdispersed, with variance exceeding the mean, which violated the model assumptions. For all models, we reported likelihood ratios for the main effect and interaction between carcass preparation temperature and developmental temperature using the *Anova* function in the ‘car’ package [44]. Once an interaction was found statistically significant (*p* < 0.05), we conducted post hoc pairwise comparisons using the *emmeans* function in the ‘emmeans’ package [45].

To examine the effect of temperatures on parental investment during pre-hatching care, we used GLMMs to analyse clutch size, carcass mass change, proportional body mass change and the roundness of prepared carcasses. In these analyses, we included temperature treatment (control/warming) as an explanatory variable. We also included the initial carcass mass (i.e. prior to breeding) and female body size as covariates, since these variables have been found to determine parental investment [46,47]. Finally, we included the family origin of the female that prepared the carcass as a random effect. For each temperature treatment, we used between one and nine individuals per family, haphazardly selected from 25 different families.

To examine the reproductive performance of burying beetles, we used GLMMs to analyse brood size, brood mass, averaged larval mass and the proportion of body mass change. The brood size was analysed with a Poisson distribution, whereas the rest of dependent variables were analysed with a Gaussian distribution. We included carcass preparation temperature and developmental temperature and their interaction as explanatory variables. We also included carcass mass and roundness as additional fixed effects to account for potential effects due to variation in the allocated carcass, and the family origin of the female that provided care as a random effect.

To examine the fitness consequences on beetle offspring, we used GLMMs to analyse the proportion of eclosion success, days to eclosion and body size of individual offspring when they became adults. We analysed the proportion of eclosion success with a binomial distribution (logit link) and days to eclosion and body size with a Gaussian distribution. For these analyses, we began by including carcass preparation temperature and developmental temperature and their interaction as explanatory variables. For the analysis of days to eclosion, we also considered the sex and body size as covariates; for the analysis of body size, we also considered the sex. Since multiple individuals could come from the same brood, we included brood ID as a random effect.

## 3. Results

### (a) Effects of elevated temperatures on pre-hatching parental care

**Figure 2.**
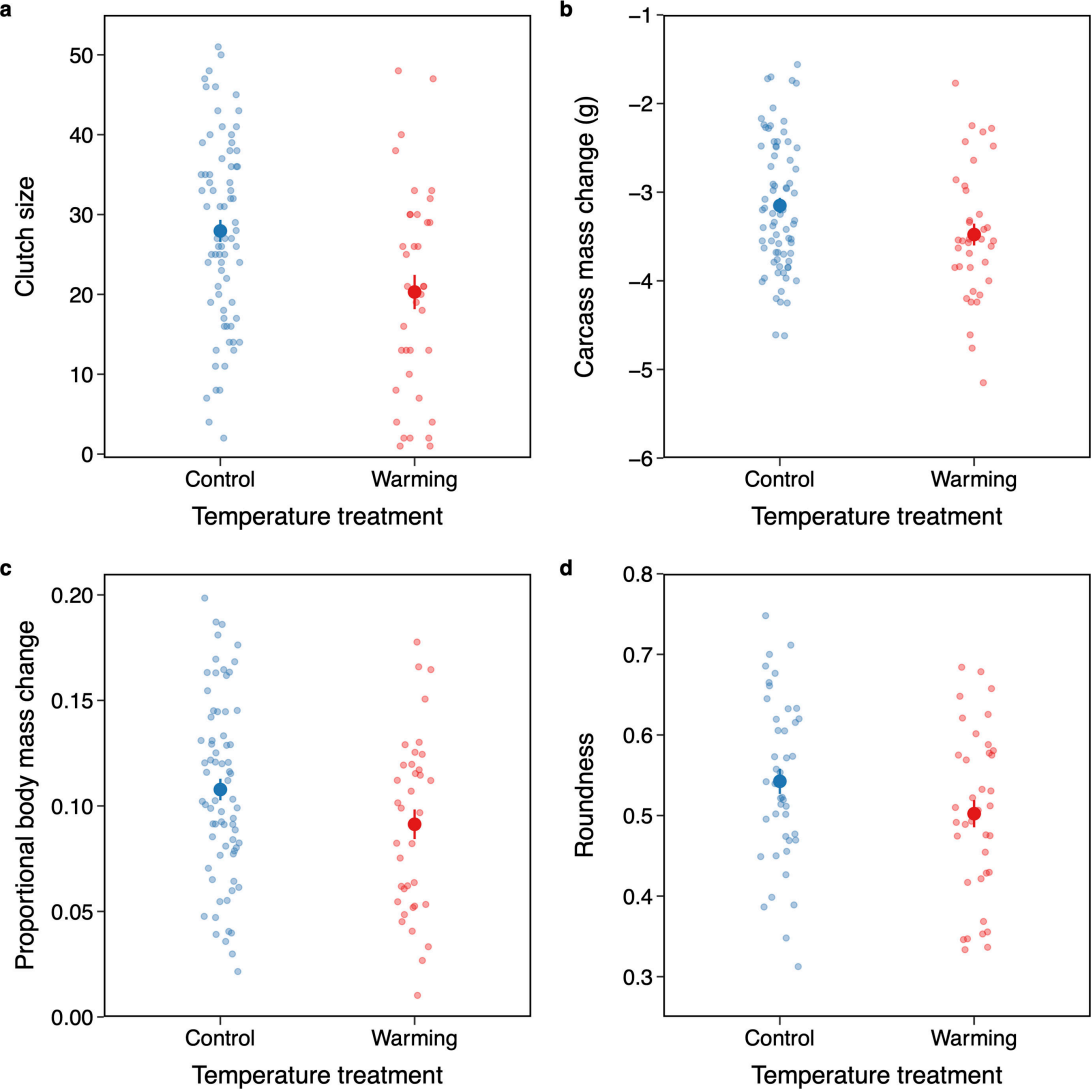
Effects of temperature on investment in pre-hatching parental care. Temperature effects on (a) clutch size, (b) carcass mass change, (c) proportional body mass change of females and (d) roundness of prepared carcass. Mean ± s.e. values are shown. Points are individual broods (*n* = 73 and 37 for control and warming conditions, respectively).

To disentangle the effects of heat stress on reproduction when exposure occurs at distinct stages of parental care, we exposed breeding females of *N. nepalensis* to contrasting control and warming conditions during the pre- and post-hatching stages and evaluated the resulting reproductive outcomes. As carcass preparation and egg-laying were completed, we found significant disruptions at the higher temperature (figure 2). Beetles produced smaller clutch sizes at warming compared to control conditions (*χ*^2^ = 5.42, d.f. = 1, *p* = 0.020; figure 2a), with none of the eggs hatched under warming conditions, consistent with our previous finding [29]. Additionally, during carcass preparation, beetles consumed more of the carcass under warming compared with control conditions (*χ*^2^ = 4.22, d.f. = 1, *p* = 0.040; figure 2b). This increased consumption resulted in a reduction in carcass weight in the warming treatment, but did not lead to an increase in beetle body mass (*χ*^2^ = 1.16, d.f. = 1, *p* = 0.282; figure 2c). The carcasses prepared under warming were also of lower quality, being less well-prepared than those under control conditions (*χ*^2^ = 3.89, d.f. = 1, *p* = 0.049; figure 2d).

### (b) Effects of carcass preparation temperature and developmental temperature on reproduction

To investigate whether maternal behavioural adjustments during high-temperature carcass preparation buffer subsequent thermal effects on larval development, we experimentally transferred carcasses prepared by beetles under either warming or control conditions and allowed other beetles to breed upon them at these temperatures (see §2). Each female received an experimental brood of 15 newly hatched larvae from the control condition, which were reared until larval dispersal.

**Figure 3.**
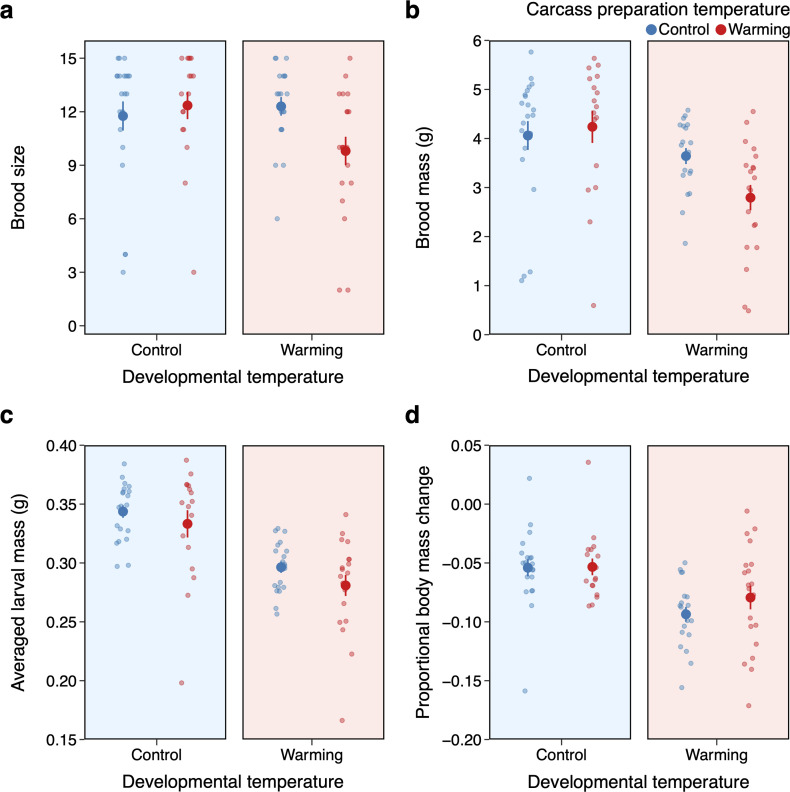
Effects of carcass preparation temperature and developmental temperature on reproductive performance. The reproductive success is measured as (a) brood size (b) brood mass, which determines the larval quality as (c) averaged larval mass. (d) Proportional body mass change is recorded as the proportional change in body mass of females from the completion of carcass preparation to larval dispersal. Mean ± s.e. values are shown. Points are individual broods (*n* = 21, 17, 20 and 20 for C_control_ – D_control_, C_warm_ – D_control_, C_control_ – D_warm_ and C_warm_ – D_warm_, respectively).

We found that carcass preparation temperature and developmental temperature interacted to affect brood size (carcass preparation temperature × developmental temperature: *χ*^2^ = 4.06, d.f. = 1, *p* = 0.044; figure 3a). While beetles assigned control and warmed carcasses produced similar brood size at control temperatures (post hoc comparison control versus warmed carcass, estimate = −0.07, s.e. = 0.10, *z* = −0.69, *p* = 0.494), beetles assigned warmed carcasses produced fewer larvae than beetles assigned control carcasses at warming temperatures (post hoc comparison control versus warmed carcass, estimate = 0.21, s.e. = 0.10, *z* = 2.10, *p* = 0.036); the effect of warming at both carcass preparation and during offspring development compounded to reduce brood size.

Similarly, brood mass was affected by the interaction between carcass preparation temperature and development temperature (carcass preparation temperature × developmental temperature: *χ*^2^ = 4.16, d.f. = 1, *p* = 0.041; figure 3b). At control temperatures, beetles had similar brood mass regardless of carcass preparation temperature (post hoc comparison control versus warmed carcass, estimate = −0.05, s.e. = 0.097, *t* = −0.53, *p* = 0.601). However, at warming temperatures, beetles had lower brood mass when assigned warmed carcasses (post hoc comparison control versus warmed carcass, estimate = 0.22, s.e. = 0.099, *t* = 2.27, *p* = 0.027), which follows given the reduction in brood size (figure 3a).

Higher developmental temperature alone, not carcass preparation temperature, resulted in lower averaged larval mass (*χ*^2^ = 36.69, d.f. = 1, *p* < 0.001; figure 3c). Providing parental care proved costly as beetles lost body mass after breeding, with proportionally greater losses when rearing young at warming temperatures compared to control temperatures (*χ*^2^ = 16.39, d.f. = 1, *p* < 0.001). Carcass preparation temperature did not significantly affect the proportional body mass change (*χ*^2^ = 1.66, d.f. = 1, *p* = 0.198; figure 3d).

### (c) Effects of carcass preparation temperature and developmental temperature on offspring at adulthood

To investigate the cascading effect of carcass preparation temperature and developmental temperature on offspring, we determined the proportion of larvae that successfully eclosed as adults, time to eclosion and their body size.

**Figure 4.**
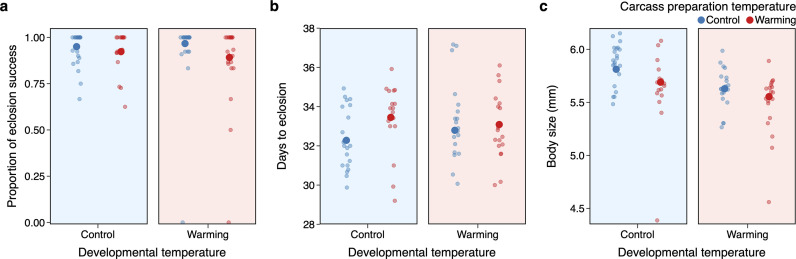
Effects of carcass preparation temperature and developmental temperature on the fitness consequences of offspring. The fitness consequences are measured as (a) the proportion of eclosion success, (b) days to eclosion and (c) body size as adults. Mean ± s.e. values are shown. For visualization purposes, data points are grouped by individual beetles from the same brood.

We found that carcass preparation temperature, but not developmental temperature, determined the proportion of eclosion success (*χ*^2^ = 3.85, d.f. = 1, *p* = 0.050), with proportionally fewer larvae successfully eclosed when feeding upon warmed carcasses (figure 4a). Of all larvae that survived to adulthood, the duration from larval dispersal to eclosion was unaffected by carcass preparation and developmental temperature (figure 4b). However, larvae feeding on warmed carcasses eclosed as smaller adults compared with those feeding on control carcasses (figure 4c).

## 4. Discussion

Elevated temperatures associated with climate change are posing significant challenges to organisms worldwide. While rapid behavioural responses to climate change through individual plasticity are now widely accepted [48], the impact of higher temperatures on the effectiveness of parental care is increasingly studied and remains equivocal. This is potentially due to a lack of understanding of which aspects of parental care are most fragile to high temperatures. To address this issue, we used the burying beetle *N. nepalensis* to mechanistically investigate the impact of heatwaves on two time periods of parental care. We found that when heatwaves only affected one aspect of parental care (pre- or post-hatching periods of care), reproductive success was equal to that of the control treatment. But when a heatwave affected both periods of care, reproductive success was lower. It is important to note that these findings are applicable only to larvae that develop from eggs to larvae under control temperatures. These results emphasize that the combined contributions of pre- and post-hatching periods of care are equally important at buffering offspring against heatwaves, highlighting the vulnerability of reproductive success to cumulative thermal stress.

Our results showed—consistent with many other studies—that temperature increase led to a reduction in reproductive investment, likely because of a greater energetic demand and/or the need for more intensive care efforts to maintain suitable conditions for offspring. Consistent with data from the congeneric *Nicrophorus orbicollis*, we show that female body mass increased when preparing a carcass and decreased after caring for offspring [49]. We note that females that provided post-hatching care all prepared carcasses in the control environment, eliminating potential carryover effects of heat stress during carcass preparation. This absence of pre-hatching heat stress likely reduced the costs females may have incurred during post-hatching parental care. Nonetheless, our results demonstrate that this trend is exacerbated by heat stress. Specifically, female mass loss was more pronounced when they reared the offspring in the warming conditions than the control, despite consuming more of the carcass under warming (figure 3d). This indicates that the heightened metabolic demands induced by heat stress may have offset the nutritional benefits of increased carcass consumption. Elevated temperatures likely increase energy expenditures through enhanced physiological and thermoregulatory processes, leaving fewer resources available for body mass accumulation. These increased energy demands may deplete the resources available for current parental care and future reproductive investment, ultimately risking the success of subsequent reproductive bouts. Although we did not directly measure parental care in this study, previous study on *N. orbicollis* found no support that warming by 4°C induced greater parental care from the caring females, indicating that females had already maximized their time in care [27].

Given the challenges posed by heat stress, should we conclude that parents have limited ability to help buffer the adverse thermal environments of the offspring? Our carcass reciprocal transplant experiment suggests that parents can partially rescue offspring from the detrimental impact of heat during the two periods of parental care we manipulated. In fact, we showed that females provided with warm carcasses to breed under control conditions and females provided with control carcasses to breed under warming conditions were both able to produce similar brood size and mass, compared with the control carcass at control conditions. However, females provided with warm carcasses were unable to effectively rear offspring under heat stress and as such had significantly smaller broods (figure 3a). Together, these findings highlight both pre-hatching care (e.g. carcass nest preparation) and post-hatching care (e.g. offspring provisioning and carcass maintenance [24]) can partially mitigate against heat stress, but also the limitation of parental care in buffering offspring against longer-term heat stress spanning throughout the breeding event.

Different aspects of parental care may be able to evolve independently in response to a change in temperature[50,51]. Our study has started to disentangle which parental care behaviours may be affected most by rising temperatures in the hope we can predict which behaviours may play a part in adaptation to climate change. Carcass preparation in burying beetles forms an essential part of pre-hatching care, which is critical for the development of their young, since it provides both a food source and a nest. Our findings showed that preparing carcasses under heat stress can be more costly than under control conditions and that the quality of carcass preparation suffers as a result. The suboptimal carcass conditions under heat stress can lead to reduced nutritional quality and increased exposure to competing microbes [52], negatively impacting offspring development and survival (figure 4a,b). Indeed, the effects of warming in our study were not limited to direct effects on larval development and survival within the experimental period, but extended even further until the offspring eclosed to adulthood. Specifically, larvae that developed on warmed carcasses, but not warming environments, struggled to successfully eclose (figure 4a); and even if they survived, they eclosed as smaller adults (figure 4c), which can subsequently reduce their fitness advantages during future breeding attempt. Other possible mechanisms of adaptive behavioural plasticity, such as carcass burial, may also help buffer offspring against thermal stress, particularly under climate warming. By burying carcasses deeper, the parents provide a cooler and more stable environment for the larvae to develop, compared with those bury carcasses closer to the soil surface. This adaptive behavioural plasticity has been found in dung beetles [53], and given its similarity with burying beetles in burial behaviour, future studies may determine whether parental care in burying beetles also allows for such adaptive response to temperature variations. Thus, we highlight the need to tease apart different components of parental care and their separate roles in shaping short- and long-term fitness consequences to better understand how species that care for their offspring will respond to a rapidly changing climate.

## Data Availability

Data and relevant code for this work are stored in Zenodo [54]. Supplementary material is available online [55].
